# Combining Metal(loid) and Secondary Metabolite Levels in *Olea europaea* L. Samples for Geographical Identification

**DOI:** 10.3390/foods13244017

**Published:** 2024-12-12

**Authors:** Raffaello Nardin, Gabriella Tamasi, Michele Baglioni, Giacomo Fattori, Amedeo Boldrini, Rodolfo Esposito, Claudio Rossi

**Affiliations:** 1 Department of Biotechnology, Chemistry and Pharmacy, University of Siena, Via Aldo Moro 2, 53100 Siena, Italy; michele.baglioni@unisi.it (M.B.); giacomo.fattori@student.unisi.it (G.F.); amedeo.boldrini@student.unisi.it (A.B.); rodolfo.esposito@unisi.it (R.E.); claudio.rossi@unisi.it (C.R.); 2 Centre for Colloid and Surface Science (CSGI), University of Florence, Via Della Lastruccia 3, 50019 Sesto Fiorentino, Italy

**Keywords:** olive, traceability, ICP-MS, HPLC, metabolites, PCA-LDA

## Abstract

To fight counterfeits, and to protect the consumer, the interest in certifying the origin of agricultural goods has been growing in recent years. In this context and to increase the accuracy of zoning models, multiple analytical techniques must be combined via a multivariate approach. During the sampling campaign, leaves and fruits (olives or drupes) were collected from multiple orchards and farms. By means of HPLC-DAD, metabolite levels were evaluated and combined with the trace and ultra-trace metal/metalloid levels evaluated by ICP-MS (QqQ). The combined dataset was then used to develop a model for geographical traceability. Furthermore, the mineral content of the soil, evaluated by means of ICP-MS, was correlated with both the mineral content in the leaves and drupes and the metabolomic profiles to further investigate the connection between the orchard’s location and characteristics of the final products.

## 1. Introduction

*Olea europea* L. is an evergreen plant cultivated for its fruits (here referred interchangeably as olives or drupes) for thousands of years [1] throughout all the Mediterranean basin, in particular for the edible oil obtained by pressing the drupes. Olive oil is an integral part of the Mediterranean diet [2] and has been proven to display health benefits including anti-inflammatory effects and play a relevant role in heart disease prevention [3,4,5,6,7] due to the high concentration of polyphenols. Their total concentration varies with cultivar, growing location and ripeness [8], but a concentration of up to 70 mg of polyphenols per 100 g can be found in drupes [9] which contribute to the unique aroma profile of the olive oil [6,10]. Cold-extracted oils are usually higher in quality and richer in natural oxidants and vitamins, such as Extra Virgin Olive Oil (EVOO). Its increasing demands, perceived benefits for the consumer and higher cost of production makes it a product frequently subject to falsification [11].

In this context, the part of the Italian PNRR-founded Agritech Center (Centro Nazionale per le Tecnologie dell’Agricoltura) that focuses on the quality and traceability of agricultural goods strives to increase the accuracy of existing methods and to reduce the zoning range. Furthermore, a growing market for high-quality, mono-cultivar EVOO brings the need to not only identify the geographical origin of these agricultural goods, but also the exact composition and cultivar used in production. Multiple approaches are available for these efforts: indeed, since secondary metabolites are known to vary with the geographical location and cultivar [8,12,13], HPLC has been used extensively in the characterization of EVOOs from different geographical regions [14,15,16]. Both targeted [17,18] and untargeted [18] analysis of secondary metabolites have been successfully used in the creation of models using multivariate statistics for the identification of geographical origin and cultivar. Similarly, due to the natural tendencies of plants to adsorb elements from the soil via the root system [19,20,21] and the variability of trace elements in the topsoil even in very close-by regions [22,23,24], trace and ultra-trace metal(loid) levels can be exploited in zoning efforts [25,26]. Quantification of these elements is usually performed by an ICP-MS analysis [26,27,28] on the mineralized sample [29] so that the complex lipophilic matrix of the olive oil does not interfere with the analysis.

The number of PDOs recognized by the EU is growing and the complexity of the databases has as well. Currently, the DOOR (Database of Origin and Registration) recognizes more than 40 olive oil PDOs and PGIs, but as this number grows, so does the number of cases of economic fraud and false claims of geographical origin on product labels. Therefore, not only have a large number of analytical strategies been developed to certify the origin and quality of EVOO production chain products, chemometric tools have also been introduced to further help differentiate overlapping regions, with linear discriminant analysis (LDA), soft independent model of class analogy (SIMCA), partial least-squares discriminant analysis (PLS-DA) or more complex AI tools being the most helpful [30]. Multivariate approaches [31,32,33] have indeed been proven successful in the separation of samples from different countries [26,31], and to a lesser extent samples from close-by regions [32,34], relying only on the mineral content of the EVOO.

Despite the growing number of papers investigating the geographical origin of agricultural goods, there is still a need to investigate the variability in the mineral and polyphenolic content in EVOs, olives or olive leaves within geographically proximate regions.

In fact, before moving on the EVOO final product, it can be extremely fruitful focusing on the olive plant matter, i.e., leaves and drupes, exploring their connection with orchards’ soil composition, which actually ties the final product to a given geographical area. A similar approach was recently applied to the wine/grape/leaf/vineyard soil system [35], with interesting outcomes. Moreover, some effort was recently devoted to the use of polyphenols, quantified by means of HPLC-HRMS as markers for the traceability of olive oils [36], which led to further investigations in this context.

Finally, despite some attempts to use multiple techniques [37] in zoning efforts, to the best of our knowledge few authors have explored the possibilities of combining metabolomic and mineral content to reduce the zoning range. Here, this possibility is explored. Since the geological features of Tuscany soil are extremely varied [38], and mineral content of the soil from different orchards in the same province may vary as well, the 2022 Agritech Sampling Campaign focused on retrieving samples from farms in the Siena and Grosseto provinces. By collecting samples of soil, leaves and drupes, which were subsequently analyzed both by means of ICP-MS and HPLC-DAD, in this work the correlation between mineral content in the soil and in the finished product and the variability of secondary metabolites and ultra-trace metal(loid)s in *Olea europaea* L. samples across Tuscany was explored.

## 2. Materials and Methods

### 2.1. Reagents

Nitric acid (HNO_3_, 70%, re-distilled, >99.999% trace metal basis) and hydrogen peroxide (H_2_O_2_, 30%, Suprapur^®^) were both acquired from Merck KGaA (Darmstadt, Germany). Diluted (1% *v*/*v*) nitric acid was used for all dilutions and standard preparation and was obtained using ultra-pure (UP) water (18.2 MΩ cm, Direct-Pure, RephiLe Bioscience, filtered with a 0.2 µm PES filter). Multielement REEs standard (16 elements, 50 mg/L each: Sc, Y, La, Ce, Pr, Nd, Sm, Eu, Gd, Tb, Dy, Ho, Er, Tm, Yb and Lu in 2% nitric acid) and multielement “Periodic Mix” standard (33 elements, 10 mg/L each: Al, As, Ba, Be, Bi, B, Ca, Cd, Cs, Cr, Co, Cu, Ga, In, Fe, Pb, Li, Mg, Mn, Ni, P, K, Rb, Se, Si, Ag, Na, Sr, S, Te, Tl, V e Zn in 10% nitric acid—Tr. HF) were acquired from Merck KGaA (Darmstadt, Germany). Internal standard and single element (Ge, Ti, Ze, Bb, Rh, Sn, Sb) solutions were obtained by dilution of the stock solution of each metal (1000 mg/L in 2% HNO_3_) acquired from Merck KGaA (Darmstadt, Germany). Au standard (1000 mg/L in HCl) was acquired from Fluka, HClO_4_ (70%, 99.999% trace metal basis) and HCl (36%, Suprapur^®^) was acquired from Merck KGaA (Darmstadt, Germany).

Gradient grade methanol (MeOH), n-exane and formic acid were acquired from Merck KGaA (Darmstadt, Germany); tyrosol (>99%), verbascoside (>98%), nuzhenide (>95%), oleuropein (>98%), luteolin (>99%) and spigenin (>99%) standards were acquired from Extrasynthese (Genay, France); whilst hydroxytyrosol (> 98%), caffeic acid (>98%), p-coumaric acid (>98%), ferrulic acid (99%), oleacin (>90%), oleocanthal (>90%) and Llgstroside (>95%) standards were acquired from Merck KGaA (Darmstadt, Germany). Folin–Ciocalteau, Na_2_CO_3_ anhydrous and gallic acid (GA) were acquired from Merck KGaA (Darmstadt, Germany). Gradient grade ethanol (EtOH), K_2_S_2_O_8_, ABTS and Trolox^®^ were acquired from Merck KGaA (Darmstadt, Germany).

### 2.2. Sampling Procedure

**Figure 1:**
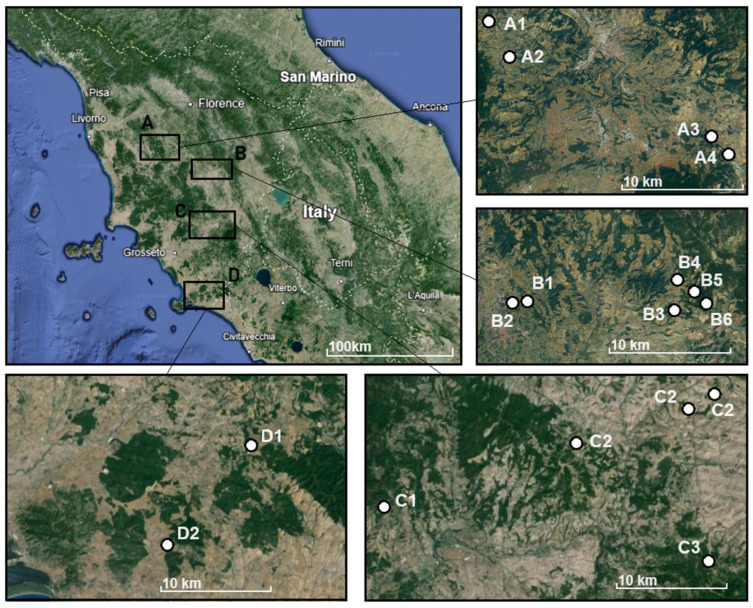
Position of the farms/orchards from where samples were collected. Zone A roughly corresponds to the Colli Senesi Region, Zone B Val d’Arbia, Zone C Val D’Orcia and Zone D Grosseto.

A total of 14 orchards belonging to 9 farms were sampled in the present work during the harvest of 2022. Most of these farms grow multiple cultivars of *Olea europea* L., and the three cultivars interest of this work (Leccino, Frantoio and Moraiolo) were all sampled when available. The location of each sampling site is reported in Figure 1. Samples were divided into four regions (A, Colli Senesi; B, Val d’Arbia; C Val d’Orcia; D, Grosseto), roughly following PDOs’ geographical regions and prior knowledge on the chemical composition of the soil from previous studies. To avoid conflicts of interests, neither the PDO geographical trade name nor the name of the farms is reported as shown in Figure 1.

Soil samples (three replicates per orchard and per variety) were collected using a stainless-steel corer: the uppermost layer of dirt (approx. 10 cm) was discarded and then a 20 cm core was retrieved to collect a portion of soil where the roots are at the densest [39,40]. Of this, the top and bottom 5 cm were also discarded to remove possible contaminants from the handling of the sample and coring. Samples were sealed in pre-cleaned PE plastic bags to avoid dust contamination.

Leaves and fruits (drupes) samples (three replicates per orchard and per variety) were collected by hand from different plants (at least three different trees to ensure a good representation of the internal variability of the orchards) and from branches that were not displaying any signs of disease or damage. At least 250 g of olives and 100 g of leaves per tree were collected. Samples were sealed in pre-cleaned PE plastic bags and then treated within the following 24 h to avoid damages.

Leaves and drupes samples were therefore washed with UP water to remove possible traces of pesticides and or dust layers, then freeze dried (−45 °C, 360 µbar) and pulverized in a blade mill (Pulverisette 11, Fritsch, Idar-Oberstein, Germany) in a liquid nitrogen (−196 °C) bath to avoid degradation of the samples due to the heat of the blades and motor. The pulverization occurred in 4 cycles of 5 s each at a speed of 10,000 rpm (3 s rest in between each cycle). Leaf samples were then sieved (500 µm) to remove debris and unprocessed leaf stems. Powdered samples were stored at −20 °C in the dark in pre-cleaned PE containers until analysis.

Soil samples were left air-drying under a flow hood in the same bag used for the collection (up to two weeks) and then grounded using an agate mortar. The resulting powder was sieved (125 µm) and stored in pre-cleaned PE containers until further processes.

### 2.3. Sample Treatment—Metabolites Extraction

Samples were analytically weighted (approx. 500 mg), and the hydrophilic components were extracted using a mixture of MeOH and UP water (80:20% *v*/*v*). Each sample was extracted with three portions of solvent (10 mL each) using an ultrasound bath (Bandelin Sonorex) followed by centrifuge to separate the samples from the solution (3 min, 4000 rpm). Some of the lipophilic compounds were also extracted during this process when treating olive samples (most likely short chain fatty acids) and they were removed with a quick clean up with 5 mL of n-hexane that was separated by centrifugation (3 min, 4000 rpm).

### 2.4. Sample Treatment—Acid Digestion

Samples were digested using an Ethos Easy microwave assisted digestor equipped with Teflon tubes. Multiple acids combinations were considered for the digestion of soil samples. Indeed, some of the elements considered such as Si and Ti, are poorly soluble in HNO_3_, with HF the most effective mineral acid for breaking the Si-O bonds in the bulk of the soil and Ti oxides needing a strong oxidant acid such as perchloric acid to fully solubilize the material. A quick experimental design (two level, full factorial) was therefore set in place before the analysis of the soil samples using CRM NIST 2710a Montana Soil, using HNO_3_ as the main acid used for the digestion and a combination of H_2_O_2_, HCl and HClO_4_ added to the digestion tube (1 mL each). The quantity of sample to be digested was also varied to gauge whether solubility was a limiting factor and a contribution to the poor recovery of some of the metals. The list of levels used in the experimental design is reported in Table S1. Each experiment was replicated three times. Due to the nature of the digestion, no true randomization of the experiment run was needed as each sample was prepared into the same digestion run. Still, each experiment run was randomly assigned to a Teflon tube to avoid any possible bias. Experimental Design (ED) analysis was performed using Design Expert Software (ver. 13.0.5.0, StatEase^®^).

For all runs, the same temperature run was used and is reported in Table 1. After each cycle of microwave assisted digestion, samples were diluted with HNO_3_ 1%, and the tubes cleaned using HNO_3_ 1% and the temperature run reported in Table 1. Recovery and ED results are reported in Table S2 and Figure S1; the analytical merits of the methods are reported in Table S3. Despite the optimization of the digestion process, some elements were found to display a rather low recovery (in some cases below 50%). This is most likely due to the exclusion of HF from the digestion process, which was performed in an effort to reduce the toxicity of the process. Indeed, every sample’s insoluble matter was left on the bottom of the digestion vessels, contributing to the overall poor recovery of some elements in the Si-rich powder.

For leaves and fruit samples, a simple acidic digestion [35,41] using HNO_3_ and H_2_O_2_ (4:1 ratio) and the temperature run reported in Table 1 have been proven to be an effective method to fully digest the majority of the elements of interest for zoning purposes with a recovery > 95% (97% on average). The efficacy of the digestion method was tested using CRM NIST 1515 (Apple Leaves) and recovery is reported in Table S4.

### 2.5. Spectrophotometric Measurements

In-depth details of the method used to estimate the total content of polyphenolic compounds (TPC) and the total equivalent antioxidant capacity (TEAC) of the samples are reported in other works [42]. Briefly, for TPC, 100 µL of sample were added to 1000 µL of UP water and 100 µL of Folin–Ciocalteu reactive. After 5 min, 300 µL of Na_2_CO_3_ 20% *m*/*v* were added and further diluted with 500 µL of UP water. Measurements of absorbance were registered at 765 nm after 30 min incubation time. A 5-point external calibration curve using gallic acid was used to estimate TPC, expressed in mg of GA equivalent per g of dry sample. For TEAC, briefly, 30 µL of samples were diluted in 70 µL of EtOH and added to 1000 µL of ABTS*^+^. Radical removal percentage was evaluated by absorbance measurements at 734 nm after 30 min of incubation time of the samples. A 5-point calibration curve using Trolox^®^ was built to extrapolate TEAC, expressed as µmol of Trolox^®^ per g of dry samples.

### 2.6. HPLC-DAD Metabolites Analysis

Samples were analyzed using a Dionex UltiMate 3000 HPLC-DAD equipped with a Kinetex 5 um Biphenyl 100A Pheomenex (100 × 2.1 mm). DAD operated in 3D field mode (min. wavelength: 190 nm, max. wavelength: 500 nm, bunch width: 1 nm) and quantification of the metabolites was done by integrating the peak at a wavelength of 220 nm.

MeOH/H_2_O acidified with formic acid (0.1% *v*/*v*) gradient run was used to separate the compounds of interest in the extracts as reported in Table 2. Eluent flow was set at 0.4 mL/min and kept constant for the separation. Examples of chromatographic separations of leaves and drupes phytochemicals are reported in Figures S2 and S3, respectively.

External 8-point external calibration curves were used for the quantitative determination of each metabolite; standard solutions were prepared by diluting concentrated (approx. 500–1000 mg/L) MeOH solutions of each compound in UP water.

### 2.7. ICP-MS Metal(loid)s Analysis

Samples were analyzed using an Agilent 8900 ICP-MS QQQ (Agilent Technologies, Santa Clara, CA, USA) equipped with Ni-Cu interfaces cones and a quartz shielded torch with a 2.5mm injector. Samples were introduced using an Agilent SPS 4 Autosampler connected to a MicroMist glass concentric nebulizer, a quartz Scott spray chamber cooled by a Peltier thermoelectric module (2 °C) to reduce water vapors in each sample. Twelve-point external calibration curves were used for the quantitative determination of each element; standard solutions were prepared by diluting concentrated (10 mg/L) standard just before analysis to avoid possible contamination. Internal standard (Ge) solution was added to both standards and samples to a concentration of approx. 20 µg/L. Most elements were analyzed in He mode, except for Li, B, Si, P and S which were also analyzed in No-Gas mode. Characteristics of plasma and sample uptake are reported in Table 3 and Table 4, respectively.

**Table 3:** Plasma characteristics and argon flow used in the analysis.

Plasma	
Plasma Mode	HMI
RF Power	1600 W
RF Matching	1.80 V
Nebulizer Gas	0.68 L/min
Makeup/Dilution Gas	0.27 L/min
Plasma Gas	15.0 L/min
Auxiliary Gas	0.9 L/min

**Table 4:** Sample uptake speed and cleanup procedure. RPS (Round per seconds) refers to the speed of the peristaltic pump.

Pre Run	
Uptake speed (Nebulizer Pump)	0.3 RPS
Uptake time	30 s
Stabilize	40 s
**Post Run**	
Rinse speed (Nebulizer Pump)	0.3 RPS
Rinse time (2% HNO_3_, 0.5% HCl)	10 s
Rinse time (1% HNO_3_)	30 s
Uptake speed (Nebulizer Pump)	0.3 RPS
Uptake time	30 s
Stabilize	40 s

### 2.8. Multivariate Analysis

Multivariate analysis was performed through R Statistical Software ver. 4.3.1 [43] using the FactoMineR package [44]. For PCA and caret package [45] for LDA. All figures were plotted using R’s package ggplot2 [46]. The tidyverse package [47] was used in data handling and statistical analysis.

## 3. Results

### 3.1. Soil Analysis

**Figure 2:**
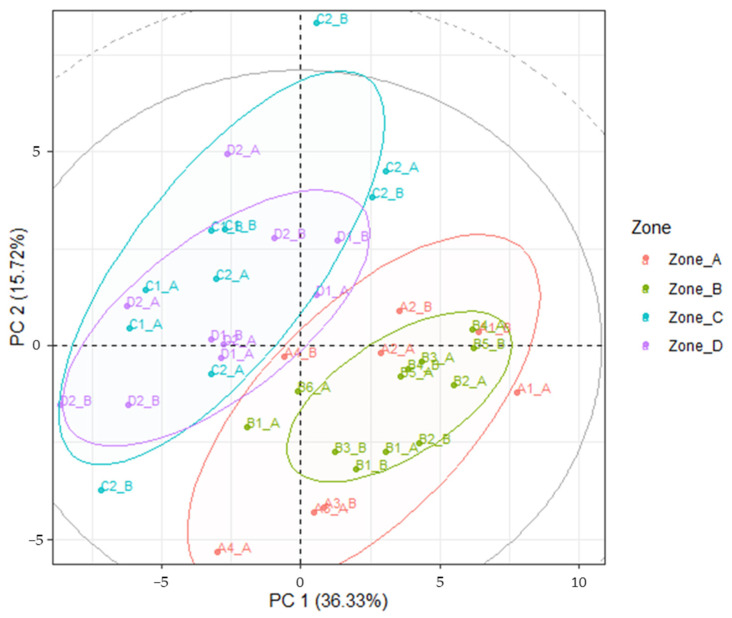
PC1 vs PC2 score projection of the soil mineral composition in the four zones considered. Ellipses are built at a critical T^2^ value at *p* = 0.05, 0.01 and 0.001.

Chemical variability, here interpreted as different mineral composition of the soil, was investigated by means of PCA. Major elements such as boron [48,49], nitrogen [50,51] and phosphorous [50,52] are known to affect plant growth but can also modulate secondary metabolite levels in plants. This is also true for non-essential metal(loid)s such as REEs [53] and heavy metals [54,55], with effects on the production of antioxidants due to the metal-induced stress [55,56]. Here, metal(loid) variability throughout the sampling area was investigated. Figure 2 reports the PCA data of all the sampling sites considered. Loadings and scree plots are reported in Figures S4 and S5, respectively.

It is worth noting that whenever multiple varieties were grown in the same orchard, samples from nearby each tree were collected and analyzed separately. This is the case for samples from the C2 (where Frantoio, Moraiolo and Leccino olive trees are grown), D2 (Leccino and Moraiolo) and D1 (Leccino and Frantoio) farms. A clear pattern emerges when considering the PC1 vs PC2 plot, with samples coming from the southern region of Tuscany (Zone C and D) clearly separated from the ones in the nearby Siena (Zone A and B), a statistically significant difference (Wilks’ = 1.8 × 10^−7^; F(105, 6.9) = 11.5; *p* = 0.0012) that could be kept in the plant mineral content and therefore exploited in the zoning efforts. This finding seems to be in agreement with the geological composition of Tuscan soil [38], even if its complex variability sometimes makes the assignment of a single geological profile to a given sampling area quite tricky.

### 3.2. Secondary Metabolites in Drupes and Leaves

**Figure 3:**
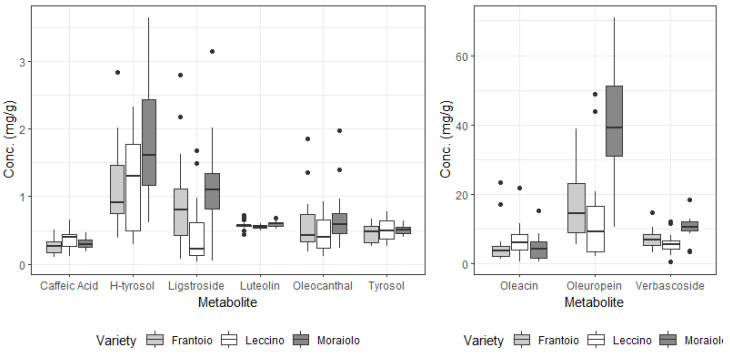
Metabolite levels (in mg/g of dried sample) found in the drupe pulps of each cultivar of *Olea europaea* L. considered.

**Figure 4:**
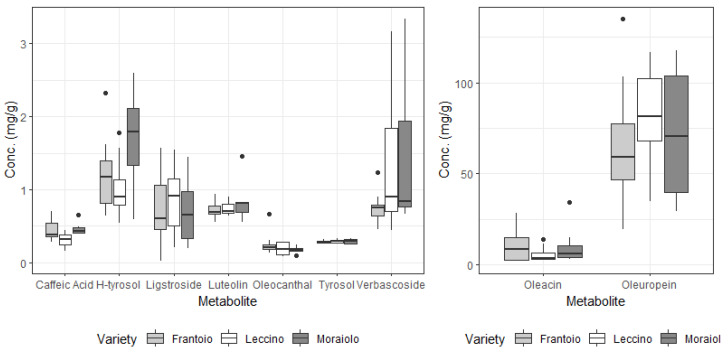
Metabolite levels (in mg/g of dried sample) found in the leaves of *Olea europaea* L. for each cultivar considered.

Metabolite levels found in both the pulp of the drupes and the leaves of olive trees considered in this work are reported in Figure 3 and Figure 4, respectively. The analytical merits of the method are reported elsewhere [42] but it is worth noting that below-LoD metabolites (apigenin, 0.19 mg/L; ferrulic acid, 0.13 mg/L; acid p-coumaric, 0.75 mg/L and nuzhenide 0.21 mg/L, each estimated as 3.3 σ/S where σ is the standard deviation of the response and S the slope of the calibration curve) were not reported and were not considered in the following analysis. Statistically significant differences (see Table 5) were found when considering most of the secondary metabolites analyzed, with only oleacin, oleocanthal and tyrosol levels not being tied to the cultivar of *Olea eureupaea* L. (ANOVA, α = 0.05) despite the fact that these compounds share metabolic pathways [50].

On the other hand, when considering the different sampling areas (see Table 5), only ligstroside, caffeic acid and oleacin (even though with a borderline statistical significance) levels show a correlation with the location of the orchards. Samples were collected in a relatively short period (less than two weeks), which leads to excluding possible variation in the ripening of the drupes influencing the results. Furthermore, despite differences in polyphenols being tied to abiotic factors and therefore geographical location [57] it must be stressed that all sites are extremely close together (approx. 30 km from one another), making zonation efforts particularly complex.

**Figure 5:**
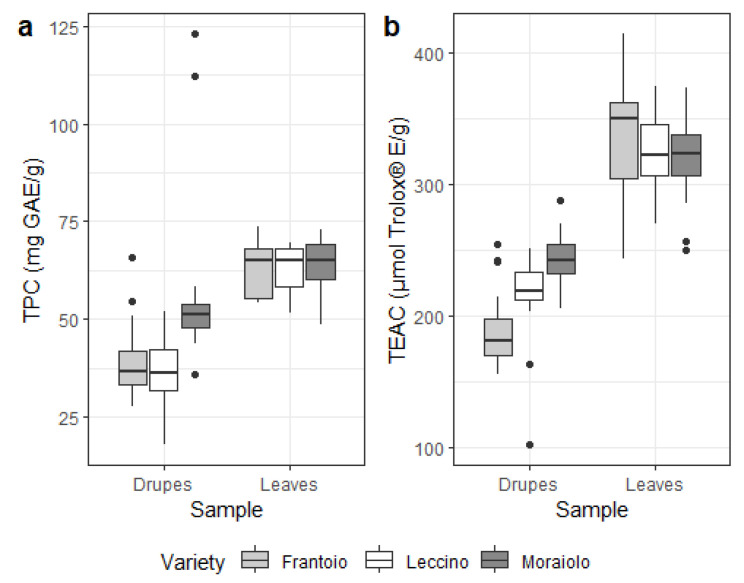
Spectrophotometric assays evaluating the TPC (**a**) and TEAC (**b**) results in samples of leaves and drupes of *Olea europaea* L. for each cultivar considered.

Similar considerations can be made for TPC and TEAC, whose levels are reported in Figure 5. Due to oleuropein and hydroxytyrosol being two of the strongest antioxidant compounds found in EVOOs and therefore in the drupes [58,59,60], and their concentrations being amongst the highest between the compounds considered, a trend emerges, similar to what was observed for single metabolites. Indeed, both TPC and TEAC are strongly related to the cultivar, but differences in climate and abiotic factors between the four considered regions are most likely not stark enough to force a different response in plants, so that no statistically significant differences are observed when looking at the four selected zones.

The same analysis was performed on the data collected for olive leaves (see Table 6), and contrarily to what was observed for the drupe samples of all the investigated metabolites, only hydroxytyrosol and caffeic acid seem to be relevant distinguishing between the three varieties, and, when considering the geographical origin of samples, only olacein is close to the significance value (even if slightly higher, with *p* = 0.051).

**Figure 6:**
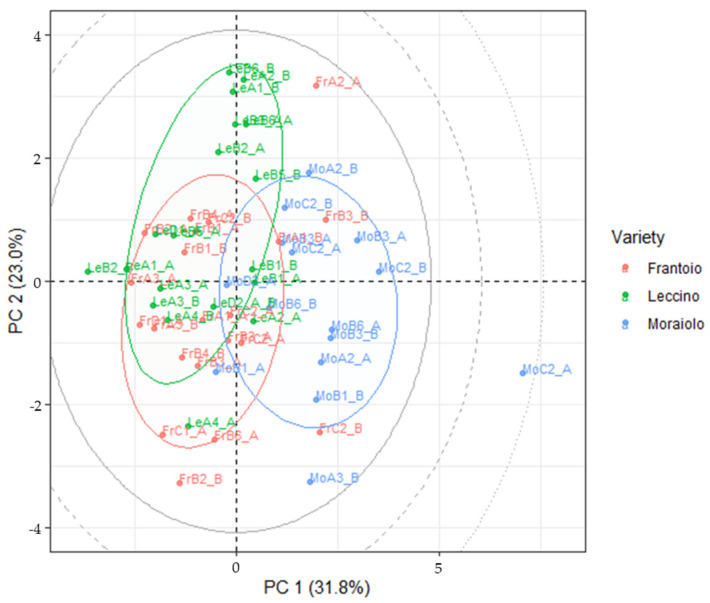
PC1 vs PC2 score projection of the secondary metabolite levels in the drupes of the three cultivars considered. Grey ellipses: critical T^2^ value at *p* = 0.05, 0.01 and 0.001.

Multivariate analysis confirms the ANOVA results. The first two components of PCA, reported in Figure 6, explaining more than 50% of the total variance of the dataset, highlighting the similitudes between samples of the Leccino and Frantoio varieties and the differences between these two and the Moraiolo ones, most likely due to the similitudes in the two cultivars when compared to the latter [61,62]. These findings were in perfect agreement with previous results obtained through a discriminant analysis of HPLC-HRMS data on the polyphenolic profile of olive leaves of the same three varieties considered in the present work [36]. Loadings and scree plots highlighting the correlation between the metabolites are reported in Figures S6 and S7, respectively. In the case of drupe samples, this difference is significant when considering the different varieties of olive tree (Wilk’s = 0.201; F (22, 84) = 4.71; *p* < 0.01) but the significance drops when investigating the differences between zones (*p* = 0.209) and MANOVA tests confirmed that for leaves, the apparent differences found are not significant when considering both geographical origin and variety (*p* = 0.248 and *p* = 0.374, respectively).

### 3.3. Mineral Content in Drupes and Leaves

**Figure 7:**
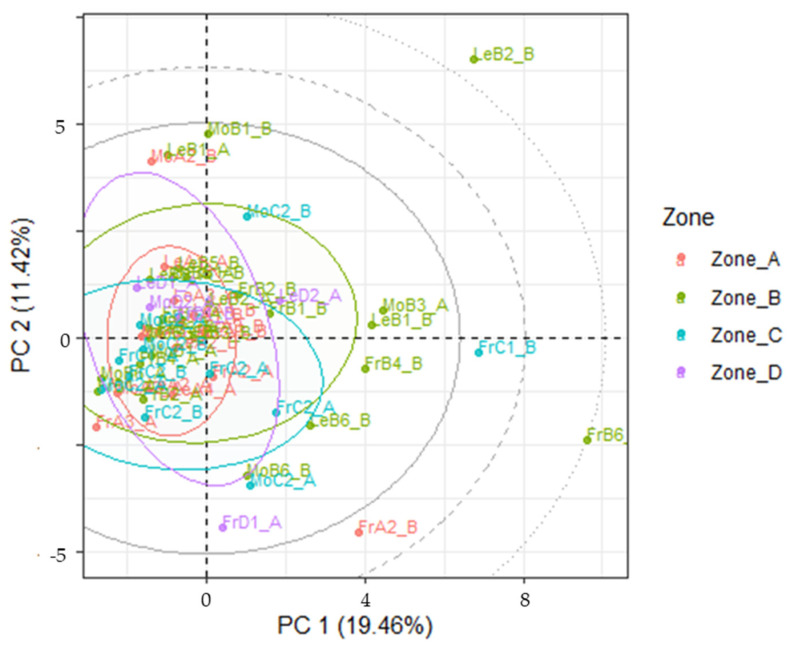
PC1 vs PC2 score projection of the mineral levels in the drupe samples in the four areas. Ellipses: critical T^2^ value at *p* = 0.05, 0.01 and 0.001.

**Figure 8:**
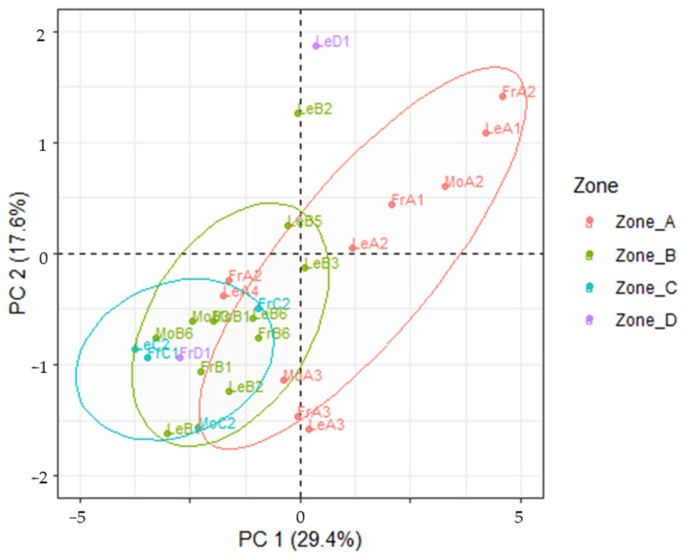
PC1 vs PC2 score projection of the mineral levels in the leaf samples in the four areas. Ellipses: critical T^2^ value at *p* = 0.05, 0.01 and 0.001.

Figure 7 reports the PC1 vs PC2 scores plot for the mineral levels inside the samples of drupes analyzed. Loadings and scree plots are reported in Figures S8 and S9, respectively. Indeed, the clear cut difference between samples coming from southerner regions of Tuscany and the one from the Siena region displayed in the soils samples is here absent, despite the MANOVA test seeming to indicate statistical differences between samples coming from different regions (Wilks = 0.0022; F (108, 54.8) = 3.35; *p* < 0.01) and trees of different variety (Wilks = 0.016; F (27, 38) = 3.67; *p* < 0.01) when considering the 36 elements selected for the analysis (elements whose concentration was below LoD in more than half of the samples were discarded). When considering the leaves samples, the number of these coming from Zone D makes it difficult to investigate the variability throughout all Tuscany, but unlike with drupes, a clearer path seems to emerge from PCA as reported in Figure 8, with PC1 separating the samples based on sea-distance. Also, this finding is in agreement with what was observed for olive leaves’ geographical origin assessed by the quantification of the polyphenolic profiles through HPLC-HRMS analyses, where the distance from the sea was identified as a key parameter influencing plants’ secondary metabolites amounts [36]. Loadings and scree plots are reported in Figures S10 and S11, respectively. Removing samples from Zone D in the drupes dataset did not improve separation.

Indeed, plants naturally adsorb metal(loid)s from the soil, both essentials [63,64] and non-essentials [64,65], but this process is dependent on the form in which these elements are in the soil and the substrate characteristic such as pH and humidity [21]. Furthermore, the distribution of the adsorbed metals in the plant is not uniform, with leaves usually collecting a higher concentration of metals [66,67]. This makes it so that a one-to-one correlation between minerals in the soil and plants is rarely present.

Indeed, when considering the metal(loid) levels in the orchards subject to study in this work, only Cs seems to display a somewhat linear correlation between the concentration in soil and the one found in the drupes, but despite this being statistically significant (*p* < 0.01), correlation is negligeable albeit positive (r^2^ = 0.388). Even when considering non-essentials minerals such as REEs, correlation is lacking with the highest degree of correlation being Pr (r^2^ = 0.0225, *p* < 0.01) and the lowest being Ce (r^2^ = 0.000352, *p* < 0.01).

### 3.4. LDA Models

Since leaves commonly only contribute to a small fraction of the material used in EVOO production (and usually they are introduced in the milling process by mistake rather than as a pondered addition) building a model able to discriminate their origin is less useful. Moreover, leaves’ mineral levels alone already provide a quite clear separation, observed in the PCA score plot (Figure 8) computed assessing their geographical origin, while in the case of drupes their metal(loid) levels were not enough to do so.

Hence, here a major interest was devoted to combining the information obtained from the two used techniques, i.e., exploiting both the metabolite and mineral content of drupes. Models were then created merging metabolite levels and mineral content as is and using the combined dataset to gauge the effectiveness of every option. The obtained models were tested both in test validation and cross validation using a randomized partition (20% of the dataset was used in validation, each class was split separately between train and test). TPC and TEAC were discarded from the LDA model due to the linearity with phenolic compound concentration [68].

To gauge the effectiveness of the models, both the cross validation and test validation of the models were repeated in multiple cycles (1500).

Metabolite levels in drupes were used directly in the creation of the model due to their limited number, but when it came to using metals levels, the number of levels compared to the number of samples made it so that some level of data manipulation on the dataset was needed. Two possible options were considered: i) removing some of the metals from the dataset until the number of levels was acceptable or ii) performing PCA on the data first to reduce dimensionality and only use the loadings in the first n PC in the making on the model. The former was discarded, as the PCA loadings plot (Figure S8) did not show any metal that contributed to the variance. Furthermore, by applying PCA first and then LDA, it should also reduce overfitting tendencies [69]. The appropriate number of PC to use in the models using the PCA-LDA approach was selected as a compromise between highest mean prediction of the model and tendencies of overfitting (as reported in Figures S14 and S15).

**Figure 9:**
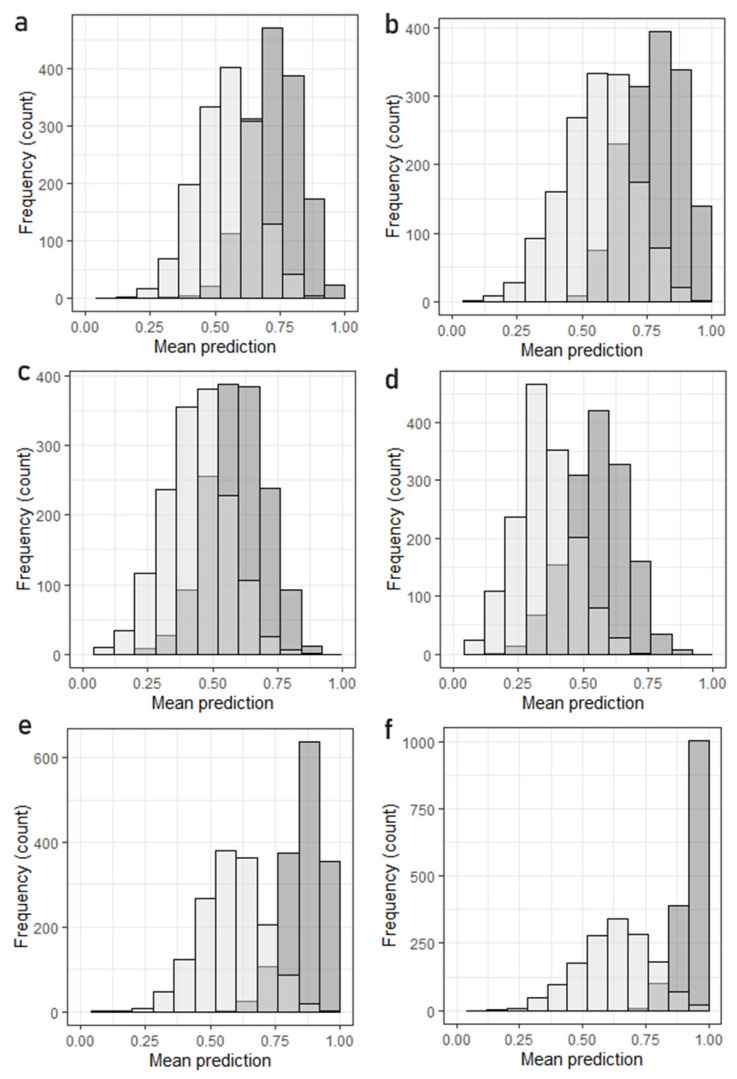
(**a**,**c**,**e**), LDA discriminating the variety of drupes; (**b**,**d**,**f**) the geographical zone. (**a**,**b**) PCA-LDA using mineral content, (**c**,**d**) HPLC metabolites, (**e**,**f**) PCA-LDA approach on both datasets. White histograms report the results of the repeated test validation, dark grey report cross validation.

Cross validation does, of course, pose the risk of overfitting the data, although it must be noted that this does not seem to be the case in any of the instances here presented. It is worth noting that combining the two datasets proved to improve the mean prediction of the model used especially when considering variety as a discriminant factor. Focusing on these LDAs, it is clear from Figure 9 how the model improves when considering the combined dataset of metal(loid)s and metabolites, both in cross- and test validation.

In particular, considering the models distinguishing geographical areas, it is clear how the use of secondary metabolites alone poses a challenge when discriminating zones very close to one another, with the model displaying a low percentage of correct predictions both in test (min. 7%, avg. 34% max. 69%) and in cross validation (min. 16%, avg. 54%, max 84%). Due to the possible direct link between minerals in the soil and plant tissue, the mineral content in drupes can be used in creating a more effective model in the discrimination of geographical origin, but a modest increase in percentage of correct predictions was found when combining the two datasets both in test (from avg. 58% to avg. 62%) and cross validation (from avg. 81% to avg. 0.92%). Table 7 summarizes the performances of the LDA tests.

More interesting are the results of the LDA model applied to the discrimination of the variety of the different samples. As it was already noted, genetic similarities between two of the species [61,62] make it difficult to use secondary metabolites in the creation of an effective model, and, indeed, the poor performances of the model both in test (min 0% avg. 45% max 91%) and cross validation (min 25% avg. 61% max 91%) are explained by the overlapping of Leccino and Frantioio samples in the LDA space. What is interesting to note is that the combined approach improves the effectiveness of the models. The tendency of mixing the two very similar varieties of olives is still somewhat present, but especially in cross validation (min 58% avg. 91% max 100%) it is clear how the combination of multiple techniques and data fusion can provide a more robust dataset and base for a discriminant model to use in the validation of origin and variety of agrifood products.

The limited dataset also provides limitations on the number of models that can be used in the discrimination of the different samples’ class. LDA is a classification technique focusing on separating samples by identifying linear functions on the dimensionally reduced dataset. A consequence of this is that samples external to the initial dataset (e.g., different varieties of drupes) will still be classified into the existing classes. Conversely, by expanding the dataset with future studies focusing not only on Tuscany orchards, but on samples coming from all throughout Italy, it may be possible to create a model capable of clustering and identifying outliers and/or samples associated with different classes.

## 4. Conclusions

Tuscany’s diverse geological terrain leads to significant variability in soil element concentrations, which can affect the elemental composition of crops grown in the region. This variability is helpful in product traceability, enhancing marketability and protecting against fraud. Using ICP-MS, trace and ultra-trace metal(loid) levels in *Olea europaea* L. leaves and drupes were analyzed and compared with orchard soil levels. Furthermore, the specific geographical area in which trees are cultivated has been proven to affect the levels of certain secondary metabolites, further providing a tool to accurately discriminate the geographical origin of certain foods and derivatives. Secondary metabolites were quantified by means of HPLC-DAD and their levels used alongside the inorganic profile to identify the olive’s cultivar, helpful due to the growing interest mono-varietal, luxury EVOOs.

It was found that soils from northern Tuscany significantly differ from those from southern Tuscany for what concerns their metal(loid) content, and this is in agreement with geological maps of this region. On the other hand, among investigated metabolites, oleacin was found as the most interesting marker to assess both for the varietal and geographical origin of drupes and leaves. This provides a new strategy to further protect consumer choice and producers of olive oil and especially EVOO which is characterized by a higher market price due to the restrictive quality parameters and the presence of specific PDOs. Rather than focusing on a single technique, multiple approaches to combine the two datasets were tested, with a combined PCA-LDA chemometric analysis on the data being the most reliable of the approaches both in the varietal and the geographical assessment, with an increase of the average correct prediction of the model of up to 30%. The use of multiple techniques not only has been proved to improve accuracy of the classification method used, but it must be noted that it also provides an additional benefit for the consumer as the product is tested on multiple levels. Indeed, not only can metabolomic profile provide important information to help discriminate adulteration in the EVOO production chain, as specific levels of polyphenols are associated with specific genus of *Olea europaea* L., but also monitoring metal(loid) levels is an integral part of one of the goals of the Agritech project, which strives for quality control. Indeed, several of the metal(loid)s used in the multivariate approach here presented are known to be toxic and their levels must be closely monitored in the finished product to ensure consumers’ safety.

Despite the limitation of the dataset, the results of this paper confirm that the study of raw matter along the whole agrifood chain can contribute greatly to traceability and quality assessment of the final product (i.e., EVOO in this case), besides deepening our knowledge on the correlation between plants’ chemistry and their geographical location. Most importantly, then, this study shows the potential of multi-analytical approaches, still quite unexplored in this context, especially when combined with multivariate statistical models. Such an integrated and synergistic approach surely represents the future of traceability and quality assessment in the agrifood field.

## Figures and Tables

**Table 1:** Temperature ramp used for the microwave-assisted digestion of soils and leaves samples. The asterisk (*) indicates a cooling step; samples were removed from the digestor once reached a temperature of 50 °C or below. The temperature ramp used for the clean-up process of the Teflon tubes is also reported.

Step	Time (min)	Power (W)	Temperature (°C)
Soil samples			
1	10	1800	160
2	15	1800	210
3	10	1800	210
4	20	0—VENT *	50 *
Leaf samples			
1	10	1800	150
2	10	1800	180
3	10	1800	180
4	20	0—VENT *	50 *
Teflon tube clean-up process			
1	10	1800	150
2	10	1800	180
3	10	1800	180
4	20	0—VENT *	50 *

**Table 2:** Gradient run used in the separation of metabolites via HPLC-DAD. The asterisk (*) indicates a linear gradient.

Time (min)	% MeOH
0.0	5.0
1.0	5.0
30.0	60.0 *
31.0	95.0 *
35.0	95.0
36.0	5.0 *
48.0	5.0

**Table 5:** ANOVA results highlighting the differences in secondary metabolite levels between drupe samples coming from different cultivar of *Olea europaea* L. and different geographical zones.

		Variety				Zone	
	F	F Crit	*p*-Value		F	F Crit	*p*-Value
Hydroxytyrosol	4.88	3.18	0.0114		2.10	2.78	0.112
Tyrosol	0.67	3.18	0.512		1.27	2.78	0.331
Caffeic Acid	5.51	3.18	0.0067		4.18	2.78	0.0101
Verbascoside	10.08	3.18	0.0002		1.78	2.78	0.161
Oleacin	0.92	3.18	0.404		2.81	2.78	0.0485
Oleuropein	20.68	3.18	2.46 × 10^−7^		1.04	2.78	0.350
Luteolin	3.66	3.18	0.0325		1.99	2.78	0.125
Oleocanthal	2.02	3.18	0.142		2.52	2.78	0.0682
Ligstroside	5.35	3.18	0.00767		4.04	2.78	0.0118
TPC	10.22	3.18	0.00018		1.70	2.78	0.178
TEAC	17.72	3.18	1.35 × 10^−6^		0.187	2.78	0.905

**Table 6:** ANOVA results highlighting the differences in secondary metabolite levels between leaves samples coming from different cultivars of *Olea europaea* L. and different geographical zones.

		Variety				Zone	
	F	F Crit	*p*-Value		F	F Crit	*p*-Value
H-Tyr	3.68	3.42	0.041		2.47	3.05	0.0897
Tyrosol	0.355	3.42	0.704		2.21	3.05	0.116
Caffeic acid	5.10	3.42	0.015		0.344	3.05	0.793
Verbascoside	1.72	3.42	0.200		2.04	3.05	0.137
Oleacin	1.03	3.42	0.372		3.02	3.05	0.051
Oleuropein	0.394	3.42	0.679		2.19	3.05	0.118
Luteolin	0.973	3.42	0.393		1.58	3.05	0.223
Oleocanthal	1.28	3.42	0.297		0.898	3.05	0458
Ligstroside	0.278	3.42	0.760		1.51	3.05	0.239
TPC	0.182	3.42	0.835		2.89	3.05	0.058
TEAC	0.451	3.42	0.643		0.473	3.05	0.704

**Table 7:** Correct predictions in test and cross-validation for the LDA models discriminating variety and geographical zone of origin of the drupes based on the metal(loid) content, secondary metabolites or a combined approach.

		Variety				Zone	
**Test Validation**	min	avg	max		min	avg	max
Metal(loid)s	0.16	0.56	0.91		0.08	0.58	1.00
Metabolites	0.00	0.45	0.91		0.07	0.34	0.69
Combined PCA	0.08	0.60	1.00		0.16	0.62	1.00
		**Variety**				**Zone**	
**Cross Validation**	min	avg	max		min	avg	max
Metal(loid)s	0.33	0.76	1.00		0.50	0.81	1.00
Metabolites	0.25	0.61	0.91		0.16	0.54	0.84
Combined PCA	0.58	0.91	1.00		0.69	0.92	1.00

## Data Availability

The original contributions presented in the study are included in the article/Supplementary Material, further inquiries can be directed to the corresponding authors.

## Supplementary Materials

The following supporting information can be downloaded at: https://www.mdpi.com/article/10.3390/foods13244017/s1, Table S1. Levels used in the ED to maximize recovery of the mineralization of soils. Figure S1. ED results, half normal plot. Table S2. Elemental mass fraction, uncertainty (95% confidence interval) and recovery percentage as found from the ICP-MS analysis of the SRM 2710a (Apple Leaves). Table S3. Limit of Detection and Limit of Quantification for each Element measured in this work as calculated as reported in the main text. Table S4. Elemental mass fraction, uncertainty (95% confidence interval) and recovery percentage as found from the ICP-MS analysis of the SRM 1515 (Apple Leaves). Figure S2. Example of chromatographic separation of phytochemicals found in leaves of *Olea europaea* L. Figure S3. Example of chromatographic separation of phytochemicals found in drupes of *Olea europaea* L. Figure S4. Loading plots for PCA highlighting the correlation between different elements in the soils of different orchards from Tuscany. Figure S5. Scree plot (a) and cumulative explained variance (b) of the PCA over the mineral levels of soils from different orchards from Tuscany. Figure S6. Loading plots for PCA highlighting the correlation between different secondary metabolites found in the drupes of different cultivar of *Olea europaea* L. Figure S7. Scree plot (a) and cumulative explained variance (b) of the PCA over the different secondary metabolites found in the drupes of different cultivar of *Olea europaea* L. Figure S8. Loading plots for PCA highlighting the correlation between different metal(loid)s levels found in the drupes of *Olea europaea* L. across Tuscany. Figure S9. Scree plot (a) and cumulative explained variance (b) of the PCA over the different metal(loid)s levels found in the drupes of *Olea europaea* L. across Tuscany. Figure S10. Loading plots for PCA highlighting the correlation between different metal(loid)s levels found in the leaves of *Olea europaea* L. across Tuscany. Figure S11. Scree plot (a) and cumulative explained variance (b) of the PCA over the different metal(loid)s levels found in the leaves of Olea europaea L. across Tuscany 600*350. Figure S12. Scree plot (a) and cumulative explained variance (b) of the PCA over the different metal(loid)s levels and secondary metabolites found in the drupes of *Olea europaea* L. across Tuscany. Figure S13. Loading plots for PCA highlighting the correlation between different metal(loid)s and secondary metabolites found in the drupes of *Olea europaea* L. across Tuscany. Figure S14: average mean prediction (black), minimum and maximum prediction (teal and orange respectively) as a function of the number of PC used in the LDA model using the mineral content in the drupes. Left, geographical zone, right, variety. Figure S15: average mean prediction (black), minimum and maximum prediction (teal and orange respectively) as a function of the number of PC used in the LDA model using the mineral and metabolites content in the drupes. Left, geographical zone, right, variety.
