# Supplementary material for: Combining Metal(loid) and Secondary Metabolite Levels in Olea europaea L. Samples for Geographical Identification
Source: Foods. 2024 Dec 12;13(24):4017. doi: 10.3390/foods13244017 (PMC11727026; doi:10.3390/foods13244017)
Supplement: Supplementary file 1 [file foods-13-04017-s001.zip › foods-3345641-supplementary.pdf]

foods

Article

Combining Metal(loid) and Secondary Metabolite Levels in *Olea europaea* L. Samples for Geographical Identification

Raffaello Nardin [1,2,*], Gabriella Tamasi [1,2,*], Michele Baglioni [1,2], Giacomo Fattori [1,2], Amedeo Boldrini [1,2], Rodolfo Esposito [1,2] and Claudio Rossi [1,2]

[1] Department of Biotechnology, Chemistry and Pharmacy, University of Siena, Via Aldo Moro 2, 53100 Siena, Italy; michele.baglioni@unisi.it (M.B.); giacomo.fattori@student.unisi.it (G.F.); amedeo.boldrini@student.unisi.it (A.B.); rodolfo.esposito@unisi.it (R.E.); claudio.rossi@unisi.it (C.R.)
[2] Centre for Colloid and Surface Science (CSGI), University of Florence, Via Della Lastruccia 3, 50019 Sesto Fiorentino, Italy
* Correspondence: raffaello.nardin2@unisi.it (R.N.); gabriella.tamasi@unisi.it (G.T.)

Abstract: To fight counterfeits, and to protect the consumer, the interest in certifying the origin of agricultural goods has been growing in recent years. In this context and to increase the accuracy of zoning models, multiple analytical techniques must be combined via a multivariate approach. During the sampling campaign, leaves and fruits (olives or drupes) were collected from multiple orchards and farms. By means of HPLC-DAD, metabolite levels were evaluated and combined with the trace and ultra-trace metal/metalloid levels evaluated by ICP-MS(QqQ). The combined dataset was then used to develop a model for geographical traceability. Furthermore, the mineral content of the soil, evaluated by means of ICP-MS, was correlated with both the mineral content in the leaves and drupes and the metabolomic profiles to further investigate the connection between the orchard's location and characteristics of the final products.

Keywords: olive; traceability; ICP-MS; HPLC; metabolites; PCA-LDA

Citation: Nardin, R.; Tamasi, G.; Baglioni, M.; Fattori, G.; Boldrini, A.; Esposito, R.; Rossi, C. Combining Metal(loid) and Secondary Metabolite Levels in *Olea europaea* L. Samples for Geographical Identification. *Foods* **2024**, *13*, 4017. https://doi.org/10.3390/foods13244017

Academic Editors: Lorenzo Strani, Caterina Durante

Received: 15 November 2024
Revised: 5 December 2024
Accepted: 9 December 2024
Published: 12 December 2024

Copyright: © 2024 by the authors. Licensee MDPI, Basel, Switzerland. This article is an open access article distributed under the terms and conditions of the Creative Commons Attribution (CC BY) license (https://creativecommons.org/licenses/by/4.0/).

Table S1. Levels used in the ED to maximize recovery of the mineralization of soils.

	Level -1	Level +1
HNO_3	5 mL	5 mL
H_2O_2	0 mL	1 mL
HCl	0 mL	1 mL
$HClO_4$	0 mL	1 mL
Smpl. mass	50 mg	100 mg

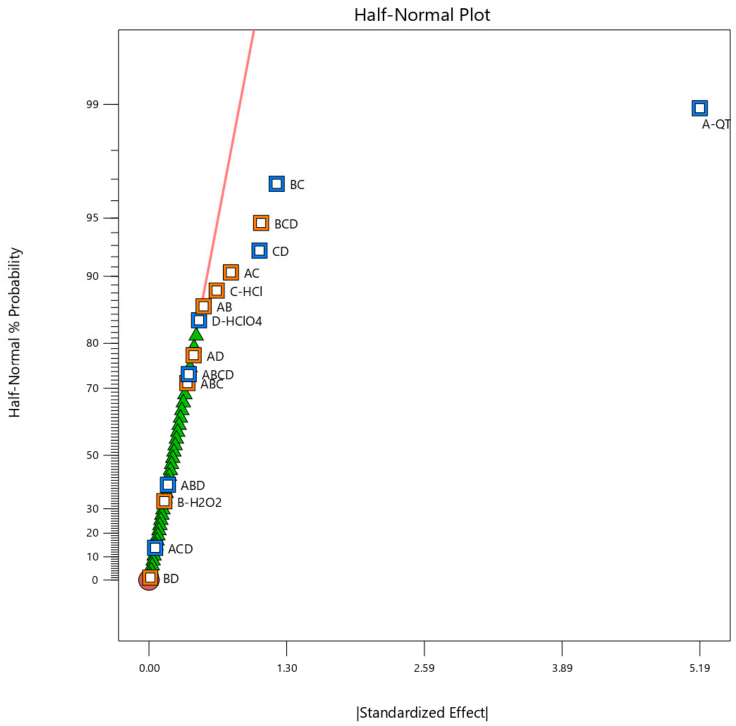

Figure S1. ED results, half normal plot. Green triangles, error estimates, blue squares, negative effects, orange squares, positive effects. Only the quantity of sample introduced in the digestor statistically influences the recovery of non-soluble metal(loid)s in Nitric acid.

Table S2. Elemental mass fraction, uncertainty (95% confidence interval) and recovery percentage as found from the ICP-MS analysis of the SRM 2710a (Apple Leaves). Reference Vaues are indicated with an asterisk (*). Non-certified elements and their respective recovery are marked with two asterisks (**). No uncertainty is provided by NIST for the non-certified mass fraction, but it's reported for completeness for the one calculated in this work.

Element	Mass	m fraction NIST (%)	m fraction (%)	Recovery (%)
**Magnesium (Mg)**	24	0.734 ± 0.038	0.354 ± 0.010	48.30
**Aluminum (Al)**	27	5.95 ± 0.05	1.88 ± 0.08	31.60
**Phosphorus (P)**	31	0.105 ± 0.004	0.0824 ± 0.0034	75.42
**Potassium (K)**	39	2.17 ± 0.13	0.745 ± 0.035	34.32
**Titanium (Ti)**	47	0.311 ± 0.007	0.108 ± 0.002	33.41
**Manganese (Mn)**	55	0.214 ± 0.006	0.153 ± 0.0019	68.68
**Iron (Fe)**	56	4.32 ± 0.08	3.52 ± 0. 8	81.62
**Copper (Cu)**	63	0.342 ± 0.005	0.298 ± 0.0048	83.50
**Zinc (Zn)**	66	0.418 ± 0.015	0.354 ± 0.0039	81.26
**Arsenic (As)**	75	0.154 ± 0.010	0.143 ±0.0019	89.15
**Lead (Pb)**	208	0.552 ± 0.003	0.479 ± 0.0084	86.73
**Element**	**Mass**	**m fraction NIST (mg/kg)**	**m fraction (mg/kg)**	**Recovery (%)**
**Boron (B)****	11	20	8	40.00
**Scandium (Sc)***	45	9.9 ± 0.1	4.1 ± 0.1	41.41
**Vanadium (V)***	51	82 ± 9	57 ± 2	66.74
**Chromium (Cr)***	52	23 ± 6	15.4 ± 0.4	67.16
**Cobalt (Co)**	59	5.99 ± 0.14	4.10 ± 0.06	68.47
**Nickel (Ni)***	60	8 ±1	6.8 ± 0.3	85.19
**Rubidium (Rb)***	85	117 ± 3	52 ± 2	44.98
**Strontium (Sr)**	88	255 ± 7	61.4 ± 1.4	24.10
**Silver (Ag)****	107	40	41	102.5
**Cadmium (Cd)**	111	12.3 ± 0.3	10.2 ±0.1	79.83
**Antimony (Sb)**	121	52.5 ± 1.6	20.4 ± 4.8	38.88
**Cesium (Cs)***	133	8.25 ± 0.11	6.08 ± 0.08	73.74
**Barium (Ba)**	137	792 ± 36	482 ± 6	58.34
**Lanthanum (La)**	139	30.6 ± 1.2	19.6 ± 0.7	61.64
**Cerium (Ce)****	140	60	38	63.30
**Neodymium (Nd)***	146	22 ± 2	14.5 ± 0.5	63.19
**Samarium (Sm)***	147	4.0 ± 0.2	2.64 ± 0.1	63.37
**Europium (Eu)***	153	0.82 ± 0.01	0.45 ± 0.01	55.73
**Gadolinium (Gd)***	157	3.0 ± 0.1	2.20 ± 0.06	73.38
**Terbium (Tb)****	159	0.5	0.3	75.00
**Dysprosium (Dy)****	163	3	1.8	60.00
**Ytterbium (Yb)****	172	2	1	50.00
**Lutetium (Lu)***	175	0.31 ± 0.01	0.134 ± 0.006	41.31
**Thallium (Tl) ***	205	1.52 ± 0.02	0.99 ± 0.03	61.98

Table S3. Limit of Detection and Limit of Quantification for each Element measured in this work as calculated as reported in the main text. Element Mass number (A) is reported in parenthesis.

Element (A)	LoD (ppb)	LoQ (ppb)	Element (A)	LoD (ppb)	LoQ (ppb)
**Li (7)**	0.18	0.60	**Mo (95)**	2.53	8.45
**B (11)**	1.24	4.14	**Ag (107)**	0.026	0.086
**Na (23)**	2.54	8.47	**Cd (111)**	0.0022	0.0072
**Mg (24)**	1.03	3.09	**Sb (121)**	0.052	0.172
**Al (27)**	2.15	7.18	**Te (125)**	0.047	0.156
**Si (28)**	-	-	**Cs (133)**	0.061	0.203
**P (31)**	10.3	34.2	**Ba (137)**	0.055	0.183
**K (39)**	-	-	**La (139)**	0.013	0.040
**Ca (42)**	18.9	62.8	**Ce (140)**	0.042	0.12
**Sc (45)**	0.31	0.93	**Pr (141)**	0.0023	0.0078
**Ti (47)**	0.11	0.37	**Nd (146)**	0.024	0.072
**V (51)**	0.023	0.069	**Sm (147)**	0.0023	0.0078
**Cr (52)**	0.030	0.099	**Eu (153)**	0.0071	0.021
**Mn (55)**	0.13	0.39	**Gd (157)**	0.014	0.042
**Fe (56)**	0.42	1.39	**Tb (159)**	0.0023	0.0077
**Co (59)**	0.049	0.15	**Dy (163)**	0.0012	0.0036
**Ni (60)**	0.035	0.114	**Ho (165)**	0.0080	0.024
**Cu (63)**	0.11	0.33	**Er (166)**	0.00040	0.0012
**Zn (66)**	0.19	0.57	**Tm (169)**	0.0090	0.027
**As (75)**	0.24	0.79	**Yb (172)**	0.00040	0.0012
**Se (78)**	5.6	16.8	**Lu (175)**	0.0018	0.0054
**Rb (85)**	0.122	0.407	**Tl (205)**	0.045	0.151
**Sr (88)**	0.073	0.21	**Pb (208)**	0.084	0.281
**Y (89)**	0.081	0.24	**Bi (209)**	0.086	0.187

Table S4. Elemental mass fraction, uncertainty (95% confidence interval) and recovery percentage as found from the ICP-MS analysis of the SRM 1515 (Apple Leaves). Non-certified elements and their respective recovery are marked with an asterisk (*). No uncertainty is provided by NIST for the non-certified mass fraction, but it's reported for completeness for the one calculated in this work.

Element	Mass	m fraction NIST (mg/kg)	m fraction (mg/kg)	Recovery (%)
**Boron (B)**	11	27.6 ± 2.8	29.7 ±2.2	107.6
**Magnesium (Mg)**	24	2710 ± 120	2585 ± 75	95.4
**Aluminum (Al)**	27	284.5 ± 5.8	257 ± 13	90.3
**Phosphorous (P)**	31	1593 ± 68	1585 ± 50	99.5
**Potassium (K)**	39	16080 ± 210	15660 ± 590	97.4
**Calcium (Ca)**	42	15250 ± 100	13750 ± 450	90.1
**Vanadium (V)**	51	0.254 ± 0.027	0.261 ± 0.025	102.8
**Chromium (Cr)***	52	0.3	0.34 ± 0.034	113*
**Manganese (Mn)**	55	54.1 ± 1.1	50.3 ± 3.7	93.0
**Iron (Fe)**	56	82.7 ± 2.6	82.4 ± 4.9	99.6
**Cobalt (Co)***	59	0.09	0.081 ± 0.003	90*
**Nickel (Ni)**	60	0.936 ± 0.094	1.035 ± 0.09	110.6
**Copper (Cu)**	63	5.69 ± 0.13	5.52 ± 0.16	97.0
**Zinc (Zn)**	66	12.45 ± 0.43	15.36 ± 2.54	123.4
**Rubidium (Rb)**	85	10.2 ± 1.6	8.4 ± 0.2	82.4
**Strontium (Sr)**	88	25.1 ± 1.1	22.9 ± 0.3	91.2
**Molybdenum (Mo)**	95	0.095 ± 0.011	0.068 ± 0.02	71.6
**Cadmium (Cd)**	111	0.0132 ± 0.0015	0.0119 ± 0.0092	90.2
**Antimony (Sb)***	121	0.013	0.010 ± 0.001	77*
**Barium (Ba)**	137	48.8 ± 2.3	46.26 ± 2.19	94.8
**Lanthanum (La)***	139	20	20.8 ± 1.3	104*
**Cerium (Ce)***	140	3	3.1 ± 0.1	103*
**Neodymium (Nd)***	146	17	17.1 ± 0.8	101*
**Samarium (Sm)***	147	3	2.95 ± 0.1	98*
**Europium (Eu)***	153	0.2	0.25 ± 0.02	125*
**Gadolinium (Gd)***	156	3	3.1 ± 0.2	103*
**Terbium (Tb)***	159	0.4	0.38 ± 0.02	95*
**Ytterbium (Yb)***	172	0.3	0.20 ± 0.05	66*
**Lead (Pb)**	208	0.470 ± 0.024	0.475 ± 0.045	101.1

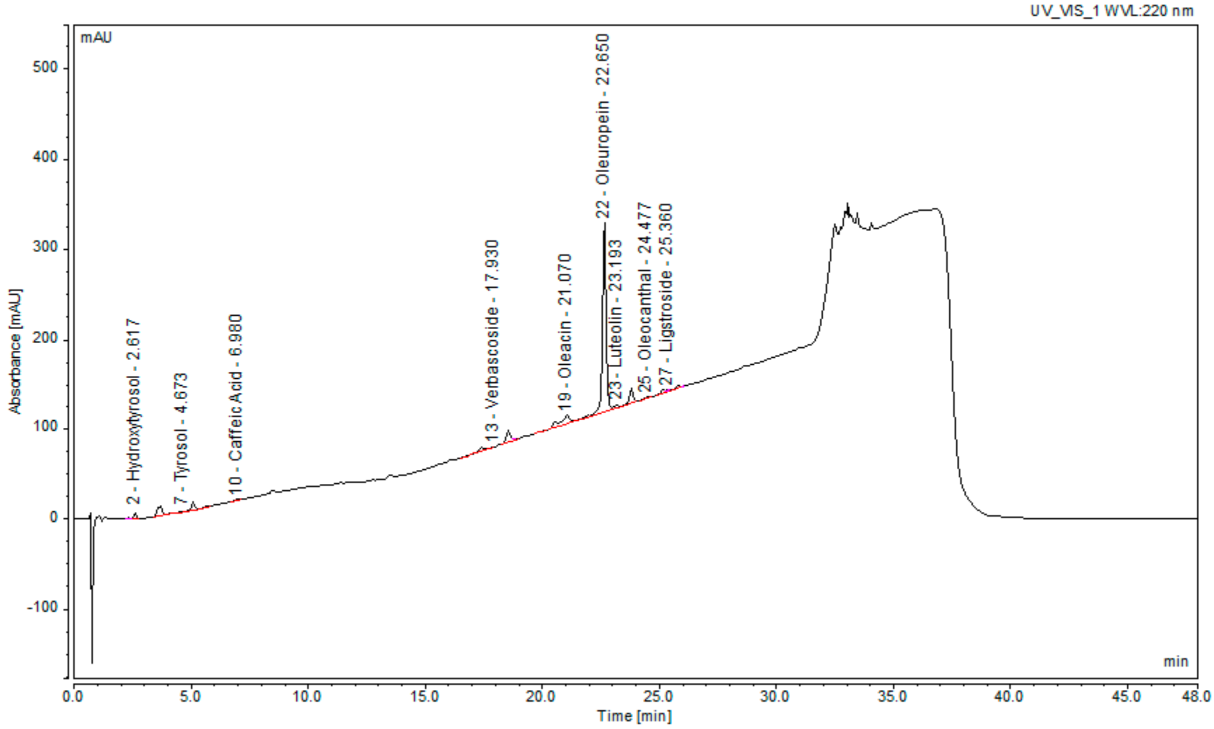

Figure S2. Example of chromatographic separation of phytochemicals found in leaves of *Olea europaea* L.

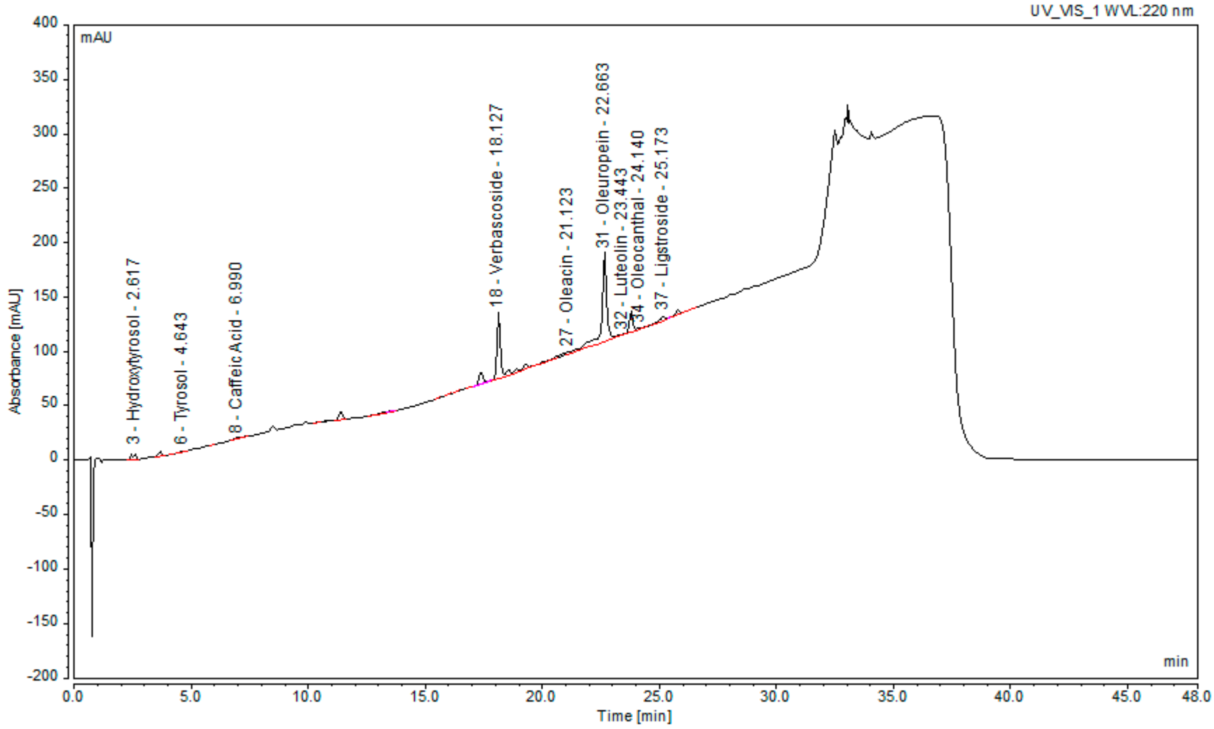

Figure S3. Example of chromatographic separation of phytochemicals found in drupes of *Olea europaea* L.

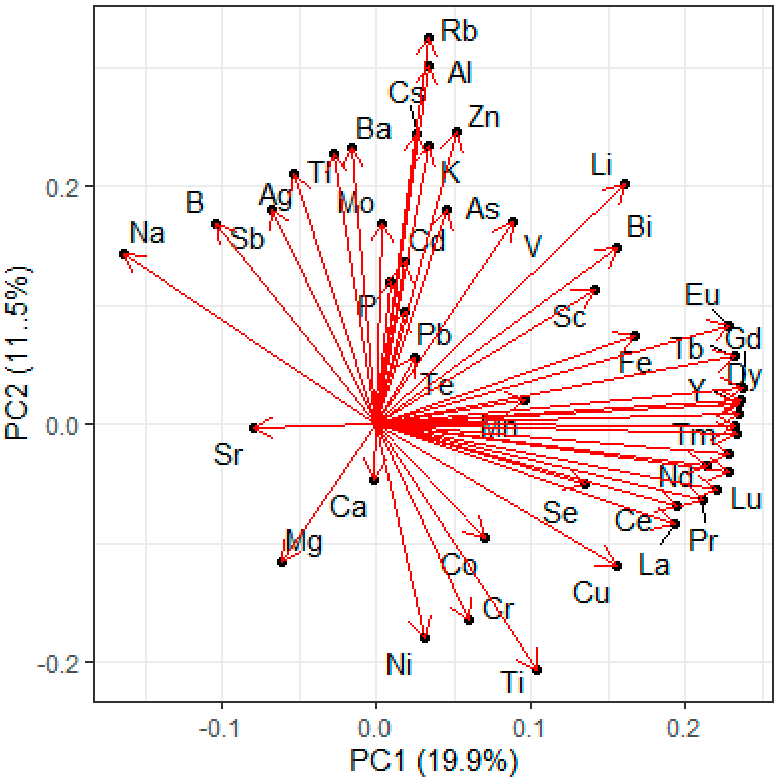

Figure S4. Loading plots for PCA highlighting the correlation between different elements in the soils of different orchards from Tuscany

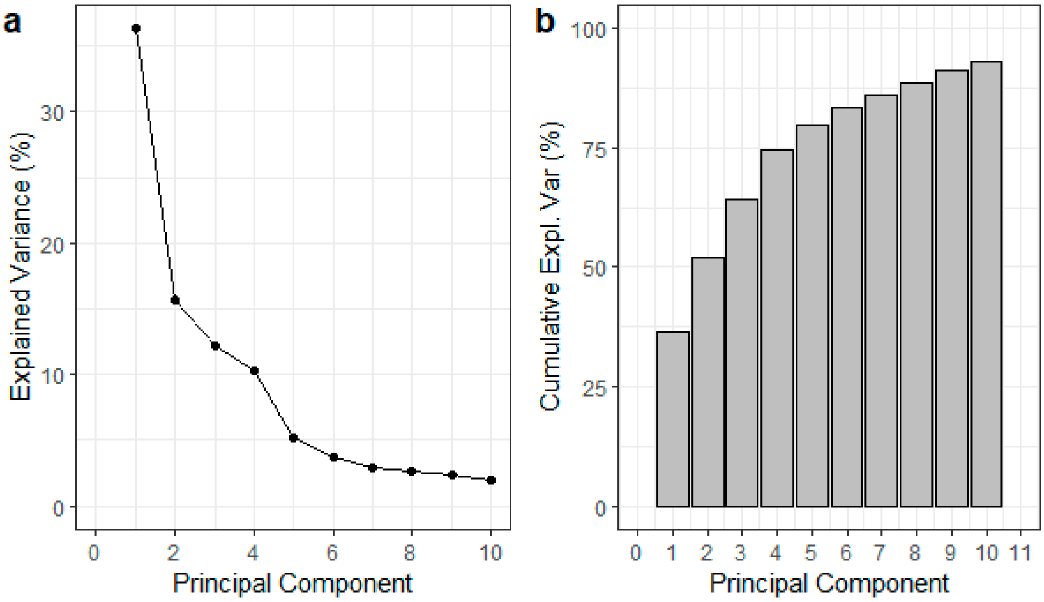

Figure S5. Scree plot (a) and cumulative explained variance (b) of the PCA over the mineral levels of soils from different orchards from Tuscany

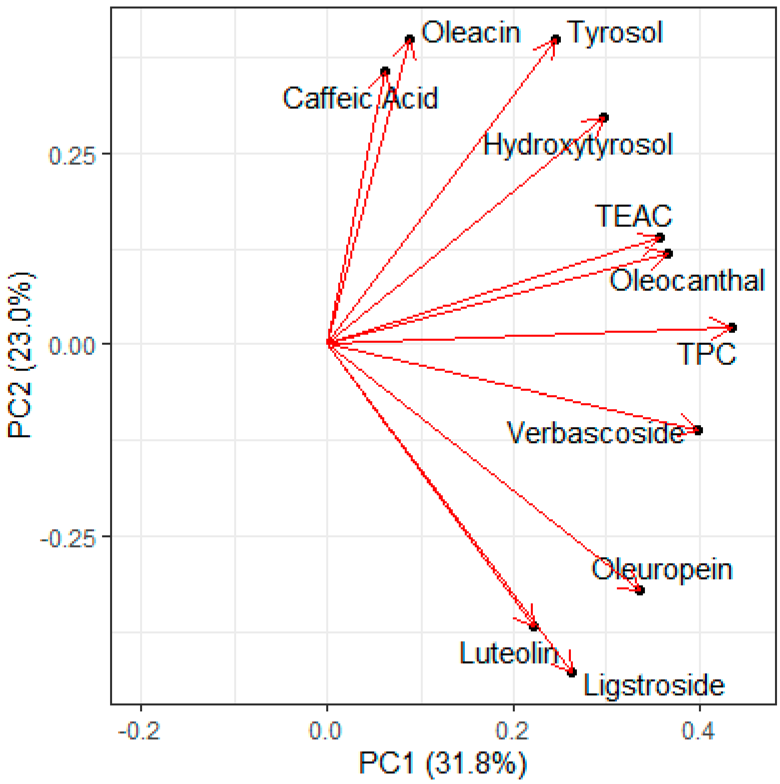

Figure S6. Loading plots for PCA highlighting the correlation between different secondary metabolites found in the drupes of different cultivar of Olea europaea L.

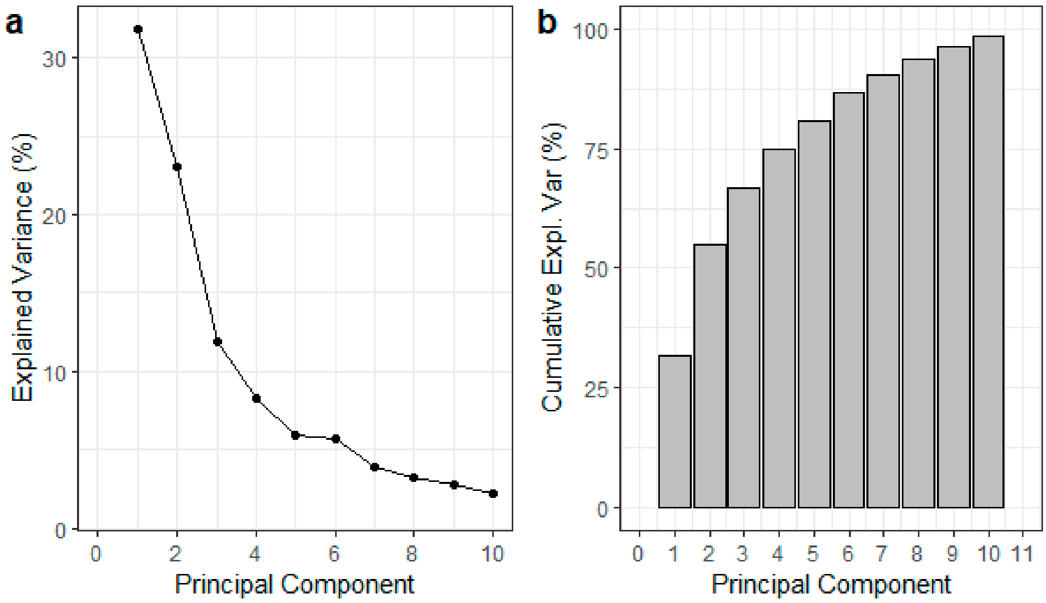

Figure S7. Scree plot (a) and cumulative explained variance (b) of the PCA over the different secondary metabolites found in the drupes of different cultivar of Olea europaea L.

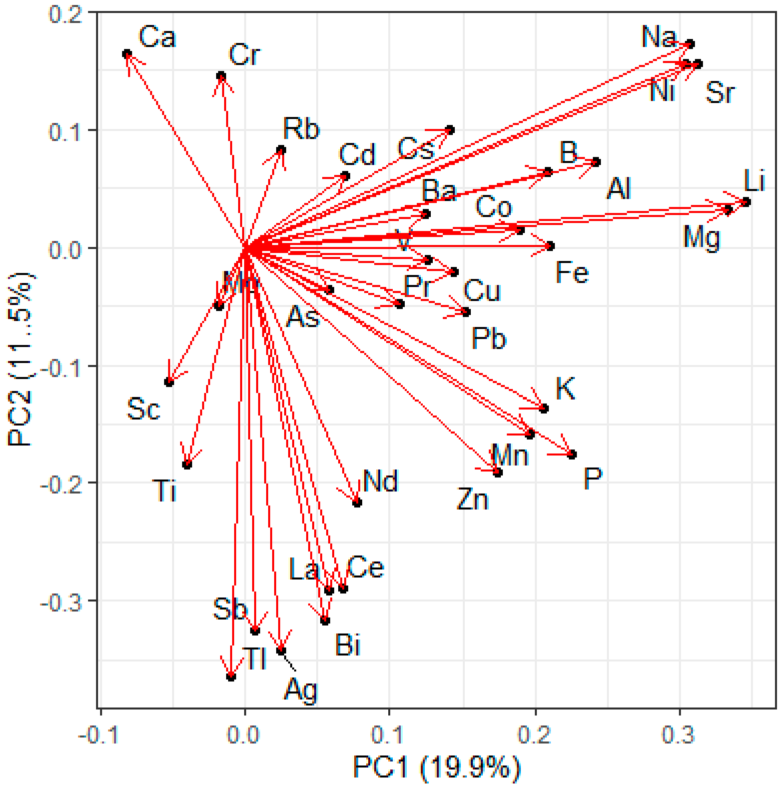

Figure S8. Loading plots for PCA highlighting the correlation between different metal(loid)s levels found in the drupes of Olea europaea L. across Tuscany

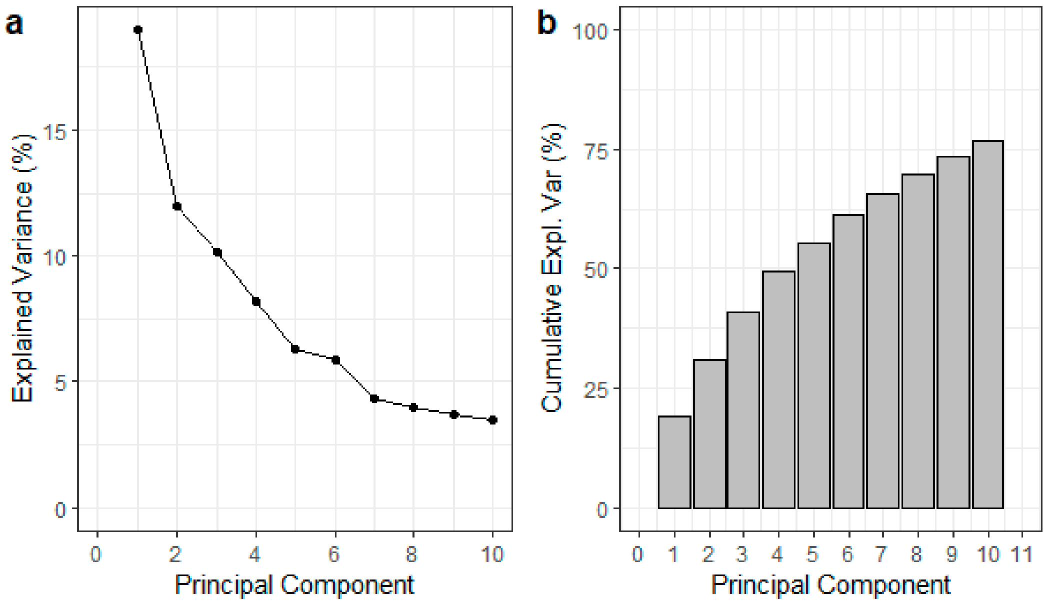

Figure S9. Scree plot (a) and cumulative explained variance (b) of the PCA over the different metal(loid)s levels found in the drupes of Olea europaea L. across Tuscany

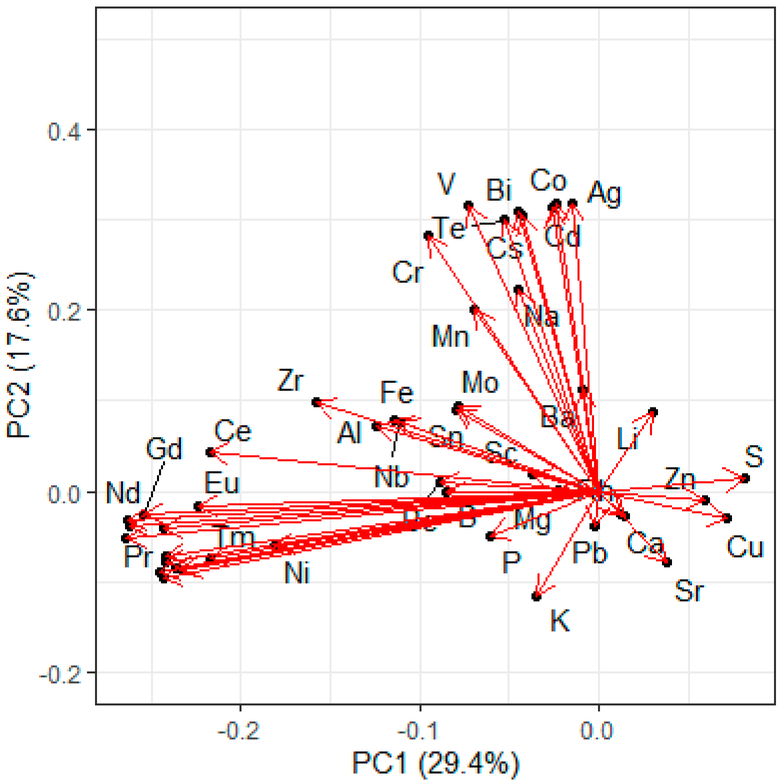

Figure S10. Loading plots for PCA highlighting the correlation between different metal(loid)s levels found in the leaves of Olea europaea L. across Tuscany

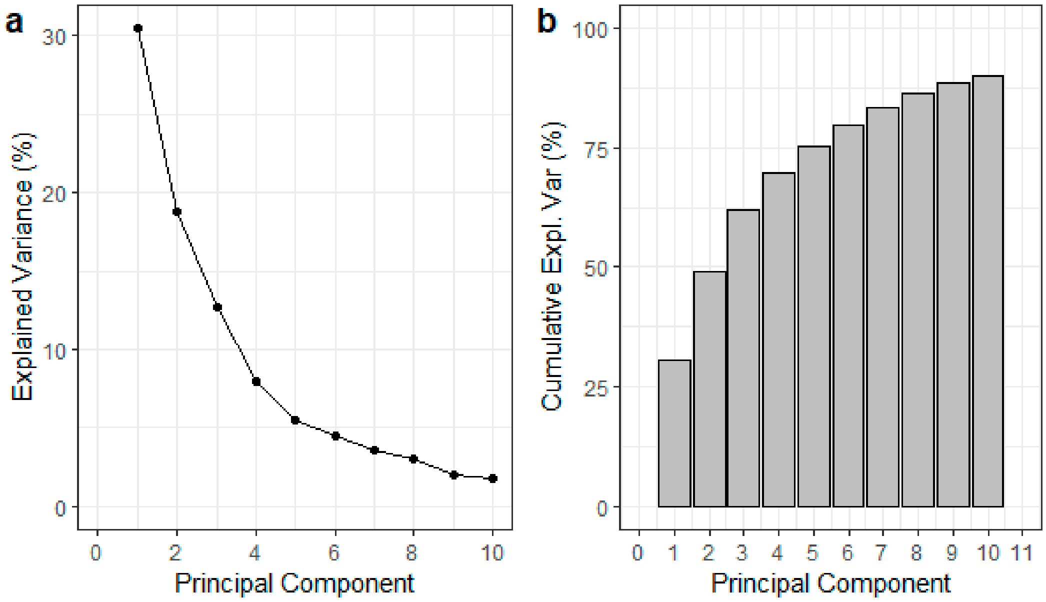

Figure S11. Scree plot (a) and cumulative explained variance (b) of the PCA over the different metal(loid)s levels found in the leaves of Olea europaea L. across Tuscany 600*350

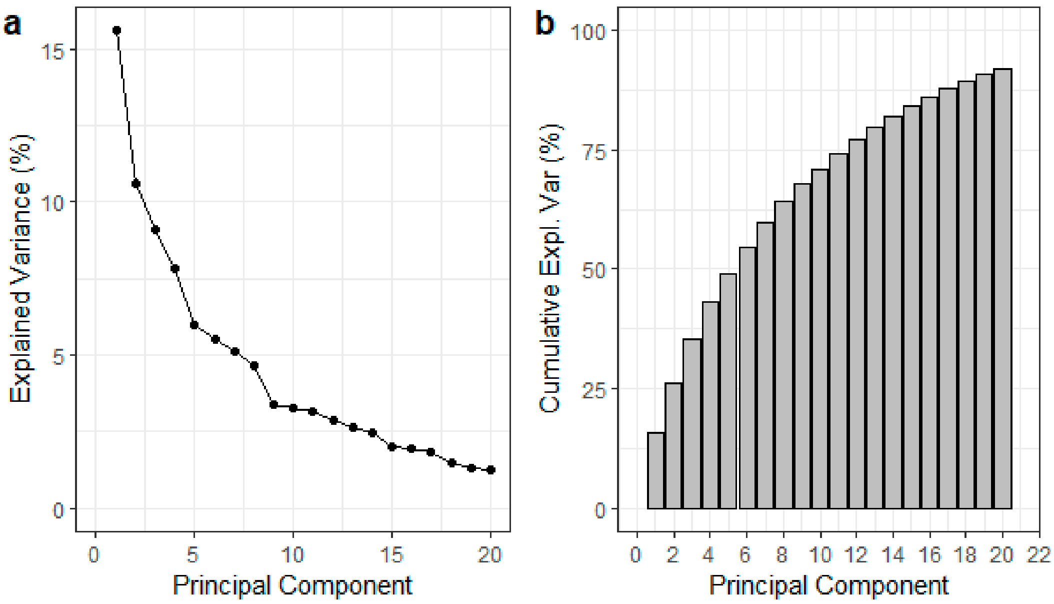

Figure S12. Scree plot (a) and cumulative explained variance (b) of the PCA over the different metal(loid)s levels and secondary metabolites found in the drupes of Olea europaea L. across Tuscany

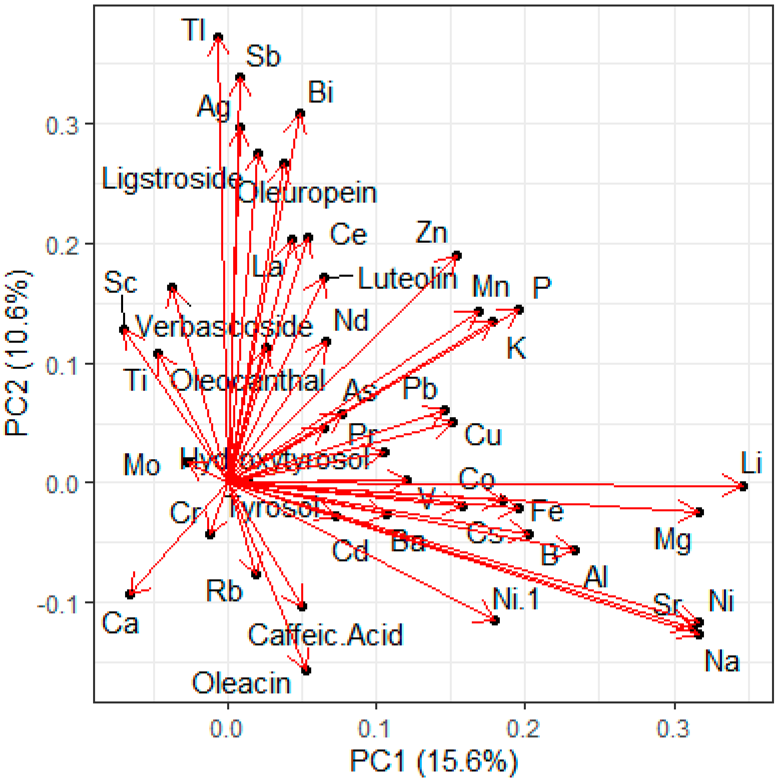

Figure S13. Loading plots for PCA highlighting the correlation between different metal(loid)s and secondary metabolites found in the drupes of Olea europaea L. across Tuscany

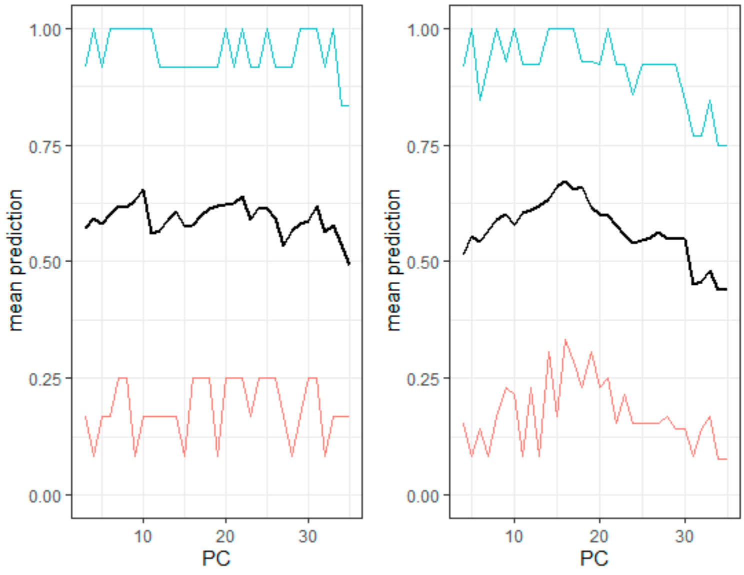

Figure S14: average mean prediction (black), minimum and maximum prediction (teal and orange respectively) as a function of the number of PC used in the LDA model using the mineral content in the drupes. Left, geographical zone, right, variety

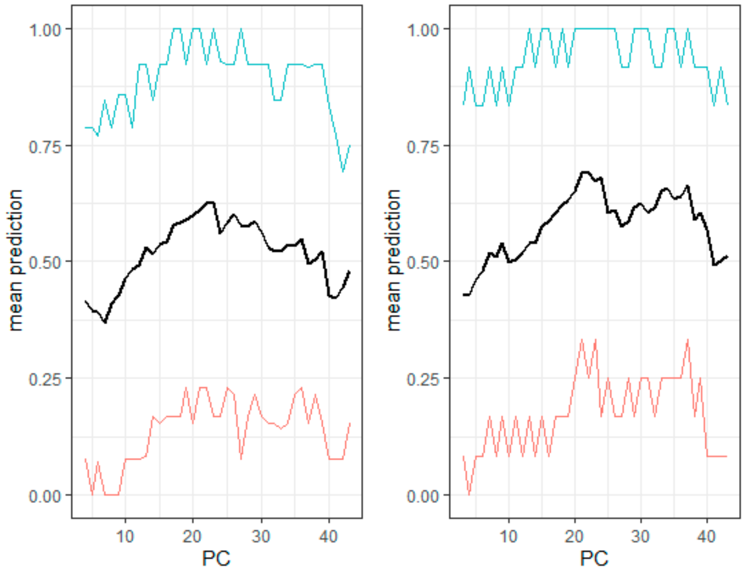

Figure S15: average mean prediction (black), minimum and maximum prediction (teal and orange respectively) as a function of the number of PC used in the LDA model using the mineral and metabolites content in the drupes. Left, geographical zone, right, variety

Disclaimer/Publisher's Note: The statements, opinions and data contained in all publications are solely those of the individual author(s) and contributor(s) and not of MDPI and/or the editor(s). MDPI and/or the editor(s) disclaim responsibility for any injury to people or property resulting from any ideas, methods, instructions or products referred to in the content.
